# Deoxycholic acid supplementation impairs glucose homeostasis in mice

**DOI:** 10.1371/journal.pone.0200908

**Published:** 2018-07-30

**Authors:** Karolina E. Zaborska, Seon A. Lee, Darline Garribay, Eumee Cha, Bethany P. Cummings

**Affiliations:** Department of Biomedical Sciences, Cornell University, Ithaca, NY, United States of America; University of Nebraska Medical Center, UNITED STATES

## Abstract

Bile acids are critical contributors to the regulation of whole body glucose homeostasis; however, the mechanisms remain incompletely defined. While the hydrophilic bile acid subtype, ursodeoxycholic acid, has been shown to attenuate hepatic endoplasmic reticulum (ER) stress and thereby improve glucose regulation in mice, the effect of hydrophobic bile acid subtypes on ER stress and glucose regulation *in vivo* is unknown. Therefore, we investigated the effect of the hydrophobic bile acid subtype, deoxycholic acid (DCA), on ER stress and glucose regulation. Eight week old C57BL/6J mice were fed a high fat diet supplemented with or without DCA. Glucose regulation was assessed by oral glucose tolerance and insulin tolerance testing. In addition, circulating bile acid profile and hepatic insulin and ER stress signaling were measured. DCA supplementation did not alter body weight or food intake, but did impair glucose regulation. Consistent with the impairment in glucose regulation, DCA increased the hydrophobicity of the circulating bile acid profile, decreased hepatic insulin signaling and increased hepatic ER stress signaling. Together, these data suggest that dietary supplementation of DCA impairs whole body glucose regulation by disrupting hepatic ER homeostasis in mice.

## Introduction

Bile acids are amphipathic steroid molecules with a well-defined role in the digestion and absorption of dietary lipid [1]. In addition, bile acids have been shown to play a critical role in the regulation of glucose homeostasis [2]. The mechanisms by which bile acids regulate glucose homeostasis remain incompletely defined. There are many different subtypes of bile acids that differ widely in their chemical composition and overall impact on health.

The variety of bile acid subtypes present within the bile acid pool is best described by reviewing the synthesis and metabolism that bile acids undergo during enterohepatic recirculation. Bile acid synthesis in the liver involves a series of enzymatic reactions during which the cholesterol sterol ring is modified and the side chain is shortened to produce the primary bile acids: cholic acid (CA) and chenodeoxycholic acid (CDCA) [3]. These primary bile acids are then conjugated with either glycine or taurine and stored in the gallbladder [4]. Primary bile acids are then secreted into the gastrointestinal tract, where they are subsequently deconjugated, dehydroxylated and oxidized in the distal intestinal lumen by gut microbes to generate the hydrophobic secondary bile acids: deoxycholic acid (DCA) and lithocholic acid (LCA) [5]. Bile acids are efficiently reabsorbed in the distal gastrointestinal tract into the portal vein and circulate back to the liver in a process known as enterohepatic recirculation [5].

Circulating bile acid profile has been implicated in the pathogenesis of insulin resistance and type 2 diabetes. However, the mechanisms responsible remain incompletely defined. In particular, the hydrophobicity index of the circulating bile acid profile is elevated in patients with type 2 diabetes compared with normoglycemic controls [6]. Different bile acid subtypes exhibit varying degrees of hydrophobicity which is determined by factors such as state of ionization and by the number, position and orientation of hydroxyl groups [7]. The relative amounts of hydrophobic versus hydrophilic bile acids determine overall bile acid profile hydrophobicity [7]. Consistent with the association between type 2 diabetes and increased circulating bile acid profile hydrophobicity index, hydrophilic bile acids subtypes, such as tauroursodeoxycholic acid (TUDCA), have been shown to improve insulin sensitivity by decreasing endoplasmic reticulum (ER) stress [8]. This suggests that bile acid profile hydrophobicity influences insulin sensitivity by regulating ER homeostasis. However, the impact of hydrophobic bile acid subtypes on *in vivo* ER homeostasis and glucose regulation has not been studied.

The ER is responsible for synthesis, folding and sorting of proteins in a cell. However, certain conditions (including increased Ca^2+^ concentration, lipotoxicity and protein accumulation within the ER) cause ER stress, which activates the unfolded protein response (UPR) [9]. The UPR is triggered by transmembrane sensors that detect unfolded proteins in the ER. These proteins are: PKR-like ER kinase (PERK), inositol-requiring enzyme 1 α (IRE1 α) and activating transcription factor (ATF) 6 [10]. Activation of the UPR is initiated to restore cellular homeostasis by decreasing protein synthesis and increasing protein degradation and autophagy [11]. Under normal unstressed conditions, binding immunoglobulin protein (BiP) binds and inactivates IRE1. However, during activation of the UPR, BiP dissociates from IRE to move to the ER lumen and help fold protein, allowing phosphorylation and activation of IRE [12]. Activation of IRE induces splicing and activation of X-box binding protein 1 (XBP1), which can then act as a transcription factor to promote synthesis of chaperone proteins [13, 14]. However, prolonged activation of the IRE-mediated pathway of ER stress signaling results in inflammation which causes insulin resistance [15]. Therefore, ER stress signaling is a critical etiologic factor in the development of insulin resistance and type 2 diabetes.

While the impact of hydrophilic bile acid subtypes on ER homeostasis and glucose regulation have been well defined, the effect of hydrophobic bile acid subtypes on these outcomes remains poorly studied. Therefore, we investigated the impact of supplementation of a hydrophobic bile acid subtype, DCA, on hepatic ER stress and insulin signaling and whole body glucose regulation.

## Materials and methods

### Animals

Male C57BL/6J mice (Jackson Laboratories, USA) were housed separately under a 14:10 hour light-dark cycle and provided with food and water *ad libitum*. At 2 months of age mice were placed on a high fat diet (HFD) supplemented with or without 0.1% DCA (Sigma-Aldrich, USA) for 3 weeks (*n* = 6 per group). High fat diet was prepared by mixing ground chow (Teklad 2018) with butter, beef tallow and soybean oil to achieve a dietary fat content of 60% energy from fat, as previously described [16]. Body weight and food intake were measured twice a week. After 3 weeks of treatment mice were fasted for six hours and tail blood samples were collected for bile acid profile measurements. Mice were then euthanized by intraperitoneal injection of 200 mg/kg pentobarbital for tissue collection. *In vivo* glucose regulation was assessed in a separate cohort of mice (*n* = 8 per group). Mice underwent an insulin tolerance test (ITT) (0.6U regular insulin/kg by intraperitoneal injection) on day 26 after dietary intervention after a 4 hour fast, as previously described [17]. Mice then underwent an oral glucose tolerance test (OGTT) (2g/kg gavage of dextrose) on day 29 after dietary intervention after an overnight fast (12 hours), as previously described [16]. During the ITT and OGTT, blood glucose was measured using a glucometer (One-Touch Ultra; Lifescan, Milpitas, CA). Mice in the second cohort were pair-fed on days 21–29 of dietary intervention to control for a trend for DCA to decrease food intake, observed in the first cohort of mice starting on day 20 after dietary intervention. Mice were then euthanized immediately after completion of the OGTT by an intraperitoneal injection of 200 mg/kg pentobarbital and tissues were collected and weighed. All animal procedures were approved by Cornell University Institutional Animal Care and Use Committee.

### UPLC-MSMS based quantitation of bile acids

Bile acids were measured from fasting mouse serum samples using ultra performance Liquid Chromatography-tandem Mass Spectrometry (LCMS/MS). Bile acids (CA, UDCA, hyodeoxycholic acid (HDCA), CDCA, DCA, LCA, α muricholic acid (αMCA), βMCA and their taurine and glycine conjugated counterparts) and the deuterated internal standard D4-CA were purchased from Sigma (St Louis, MO). All solvents for sample extraction were LCMS grade. 100μl of serum samples were spiked with deuterated internal standard at a level of 5nM and were mixed with ten volumes of acetonitrile for protein precipitation. After incubation at 4°C for 15 minutes, the mixture was centrifuged and the organic layer was transferred to a LCMS vial and evaporated under nitrogen stream. The extracted bile acids were resuspended in 55% methanol containing 5mM ammonium formate for further analysis. Calibration standards and QC samples for all the bile acids were prepared in control serum spanning a range of 0.5nM to 1000nM and extracted the same as the samples. LCMS/MS analysis was performed using an API 4000 triple quadrupole mass spectrometer equipped with an electrospray ionization source and integrated to an Eksigent UltraLC 100 system (ABSCIEX, Foster City, CA). Instrumentation control, data acquisition and quantitation were done using Analyst 1.6 software. Chromatographic separation was performed on a Phenomenex Kinetex C18 column (50x2.1mm, 1.7u, 100A) maintained at 40°C. The flow rate was maintained at 250ul/min. The mass spectrometer was operated under selective reaction monitoring (SRM) mode with negative electrospray ionization using the following optimized parameters: curtain gas: 20psi; nebulizer gas: 40psi, heater gas: 50psi, temperature 450°C, and ion spray voltage: -4500v.

### Western blotting

Immunoblotting was performed as previously described [16, 18]. Primary antibodies were obtained from Santa Cruz Biotechnology: α tubulin, 1:10000, sc-23948; BiP, 1:1000, sc-13968; XBP1, 1:1000, sc-7160; Akt, 1:5000, sc-8312; Cell Signaling Technology: pAkt, 1:5000, S473 or Abcam: IRE, 1:2000, ab37073; pIRE, 1:1000, ab48187; TNFα 1:5000 ab66579. Chemiluminescence was performed with Luminata Forte Western HRP Substrate (Millipore) and detected using Chemidoc MP Imaging system. Protein expression was quantified using ImageLab 5.1 software.

### Statistical analysis

Data are presented as mean ± SEM. All statistical analysis was done using GraphPad Prism (v6 GraphPad software, USA) by ANOVA or Student’s t-test, as indicated. *P*<0.05 values were considered statistically significant.

## Results

### DCA supplementation does not influence food intake or body weight

**Fig 1:**
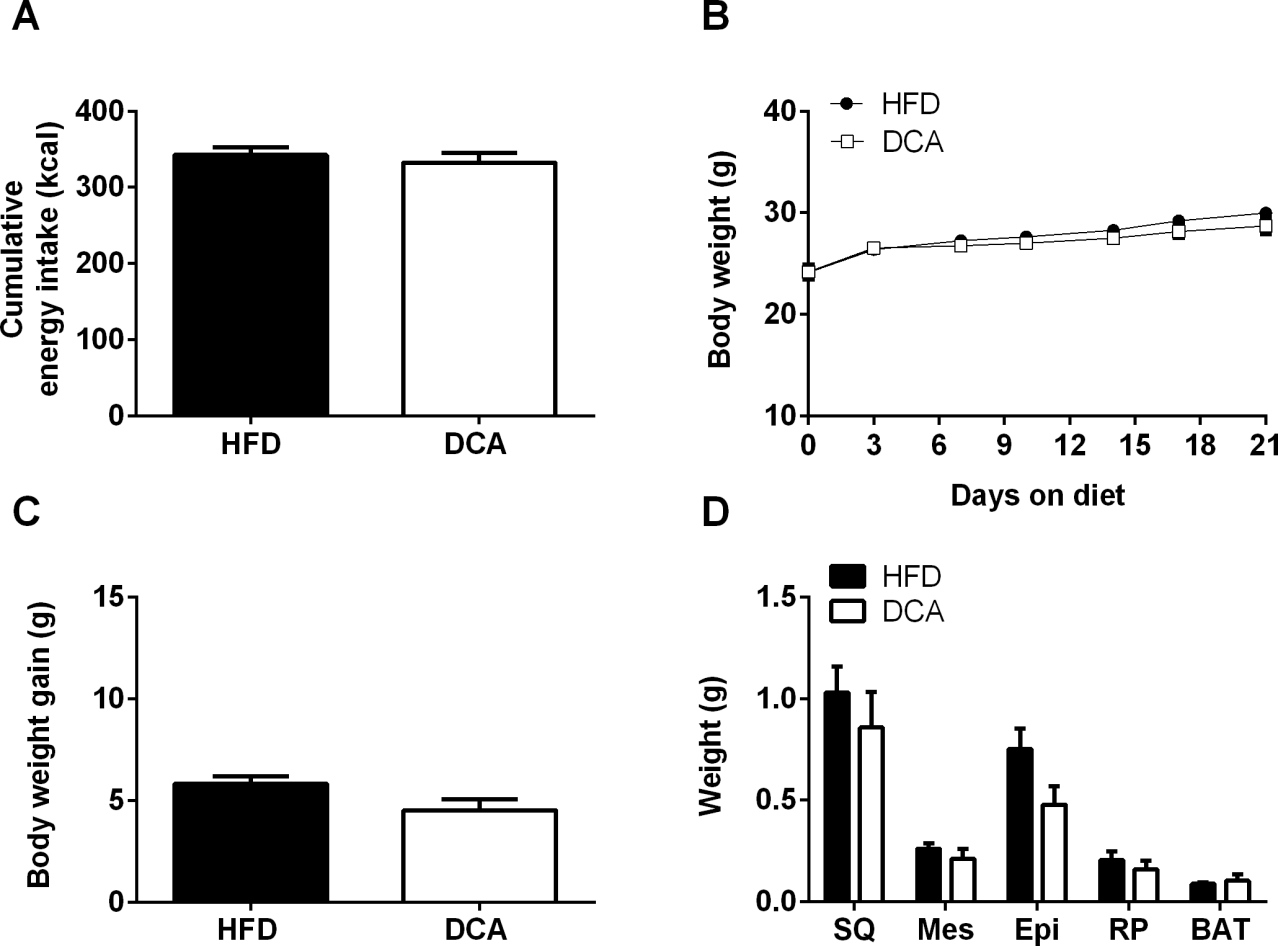
Food intake, body weight and adiposity. Energy intake (days 1–17, A), body weight (B), body weight gain (C) and adipose depot weights (subcutaneous (SQ), mesenteric (Mes), epididymal (Epi), retroperitoneal (RP) and brown adipose tissue (BAT)) (D). Data are expressed as mean ± SEM, *n* = 6 per group.

DCA supplementation did not affect cumulative energy intake, body weight, body weight change or adipose depot weight (Fig 1A–1D). Furthermore, DCA supplementation did not affect fasting serum insulin or lipid concentrations (S1 Fig). Therefore, the effects of DCA reported herein are independent of body weight, adiposity and circulating lipid concentrations.

### DCA supplementation increases circulating bile acid profile hydrophobicity

**Fig 2:**
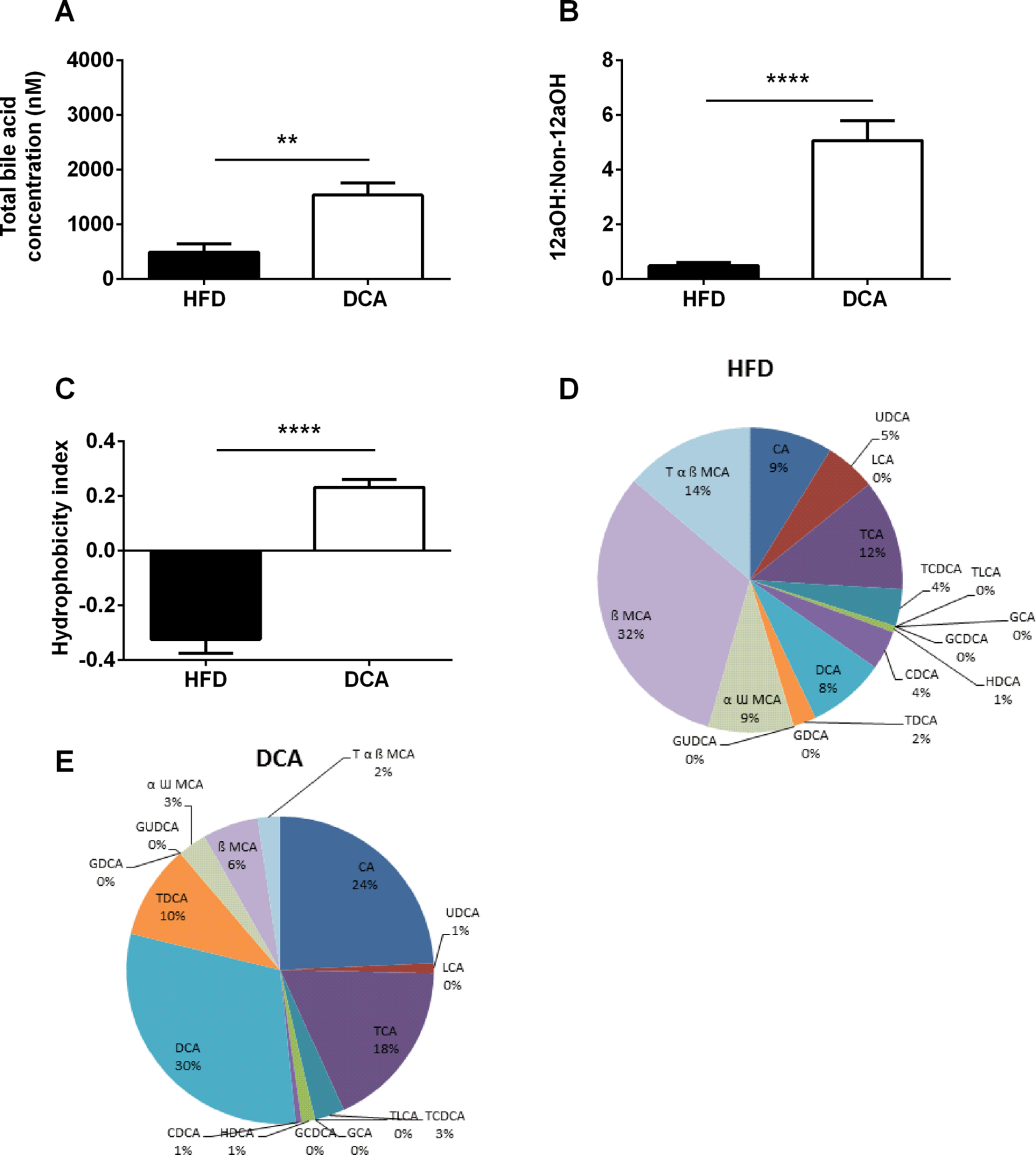
Fasting serum bile acid profile. Total bile acid concentration (A), 12αOH:non-12αOH ratio (B) and hydrophobicity index (C) in fasting serum samples. Relative proportions of bile acid subtypes in fasting serum samples from HFD (D) and DCA treated (E) mice. Data are expressed as mean ± SEM, **P<0.01, ****P<0.0001 by Student’s t-test, *n* = 6 per group. TCA, taurocholic acid; TLCA, taurolitocholic acid; HDCA, hyodeoxycholic acid; GUDCA, glycoursodeoxycholic acid; CDCA, chenodeoxycholic acid; UDCA, ursodeoxycholic acid; αω MCA, αω muricholic acid; βMCA, β-muricholic acid and Tαβ MCA, tauro-αβ muricholic acid.

Total serum bile acid concentration was increased in DCA-treated mice compared with control mice (Fig 2A, *P*<0.01). Of note, dietary supplementation with bile acids, including DCA, decreases the expression of hepatic bile acid synthesis enzymes [19, 20]. Therefore, increases in circulating bile acid concentrations in response to dietary bile acid supplementation occur independently of hepatic bile acid production. The 12α-hydroxylated (12αOH) bile acid subtypes (CA, DCA and their conjugated forms) are positively associated with insulin resistance [6]. As expected, the circulating 12αOH:non-12αOH bile acid ratio was elevated in DCA-treated mice compared with control mice (Fig 2B, *P*<0.0001). The hydrophobicity index of the circulating bile acid pool, calculated as previously described [7], was also significantly increased in DCA-treated mice compared with control mice (Fig 2C, *P*<0.0001). Consistent with these changes in bile acid profile, circulating levels of DCA, taurodeoxycholic acid (TDCA) and CA were elevated in DCA-treated mice compared with control mice, as both an absolute value and as a percentage of the circulating bile acid pool (Fig 2D and 2E, S1 and S2 Tables, *P*<0.05). The circulating levels of hydrophilic bile acid subtypes (including: UDCA, αωMCA, βMCA and TαβMCA) were decreased in DCA-treated mice compared to controls as a percentage of the total circulating bile acid pool, but not as an absolute value (Fig 2D and 2E, S1 and S2 Tables, *P*<0.05).

### DCA supplementation impairs hepatic ER homeostasis and insulin signaling

**Fig 3:**
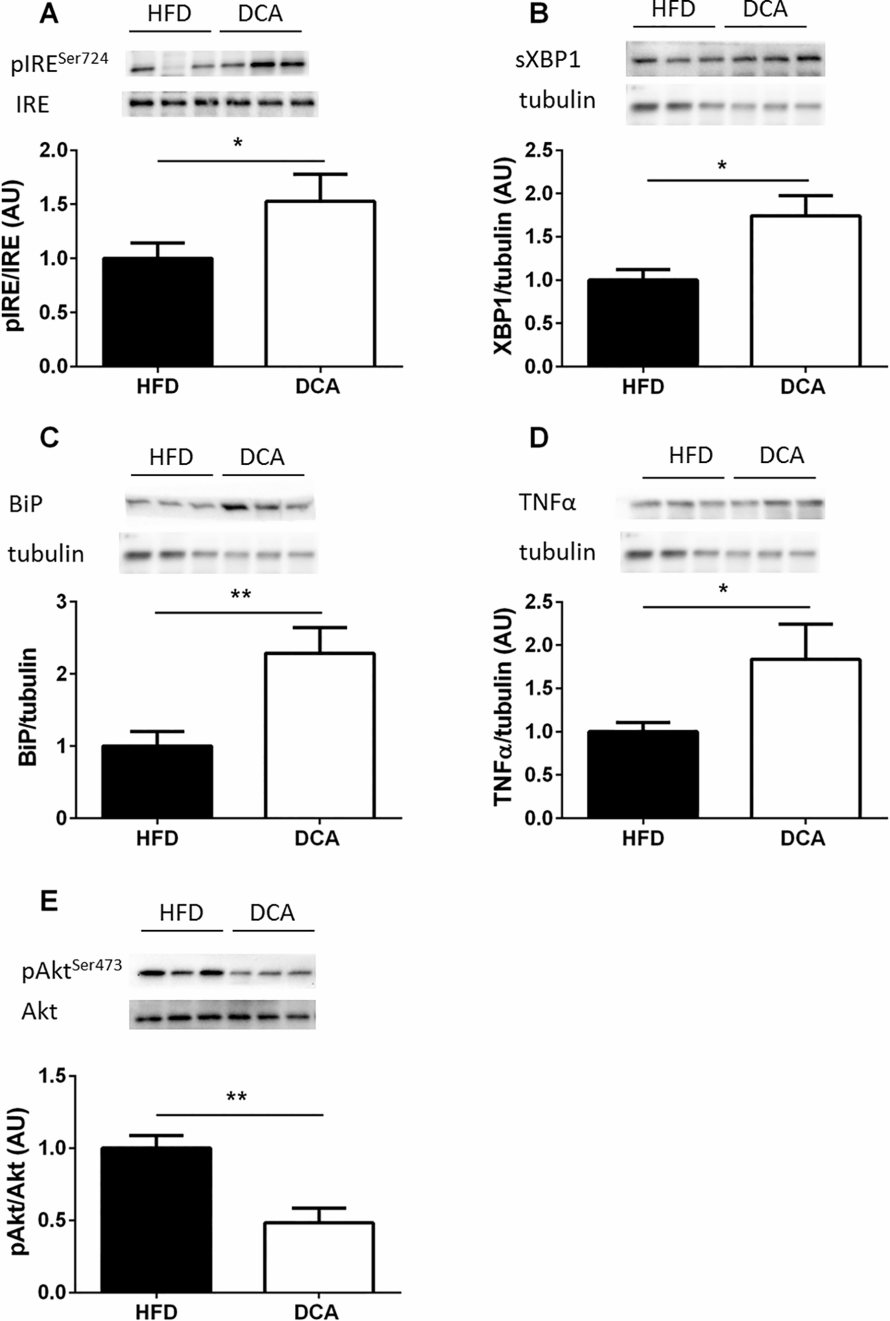
DCA increases hepatic ER stress signaling and decreases hepatic insulin signaling. Phosphorylated to total IRE (A) and spliced XBP1 (sXBP1) expression normalized to tubulin (B). BiP (C) and TNFα (D) expression normalized to tubulin and phosphorylated to total Akt expression (E). Data are expressed as mean ± SEM, *P<0.05, **P<0.01 by Student’s t-test, *n* = 6 per group.

DCA supplementation increased hepatic IRE1^Ser724^ phosphorylation and its downstream mediator, spliced XBP1 (sXBP1) expression, compared with HFD-fed control mice (Fig 3A and 3B, *P*<0.05). DCA supplementation also increased hepatic BiP protein expression approximately two-fold compared with HFD-fed control mice (Fig 3C, *P*<0.05). Increased ER stress signaling promotes insulin resistance by increasing inflammatory signaling [21]. Consistent with the increase in hepatic ER stress signaling, DCA supplementation increased hepatic TNFα expression compared with HFD-fed control mice (Fig 3D, *P*<0.05). Moreover, DCA decreased hepatic Akt^Ser473^ phosphorylation two-fold compared with HFD-fed control mice (Fig 3E, *P*<0.01). Of note, ER stress signaling is increased in response to obesity [11]. Since we did not observe differences in body weight between our groups, the effect of DCA to impair ER homeostasis and insulin signaling is independent of body weight.

### DCA supplementation impairs glucose homeostasis

**Fig 4:**
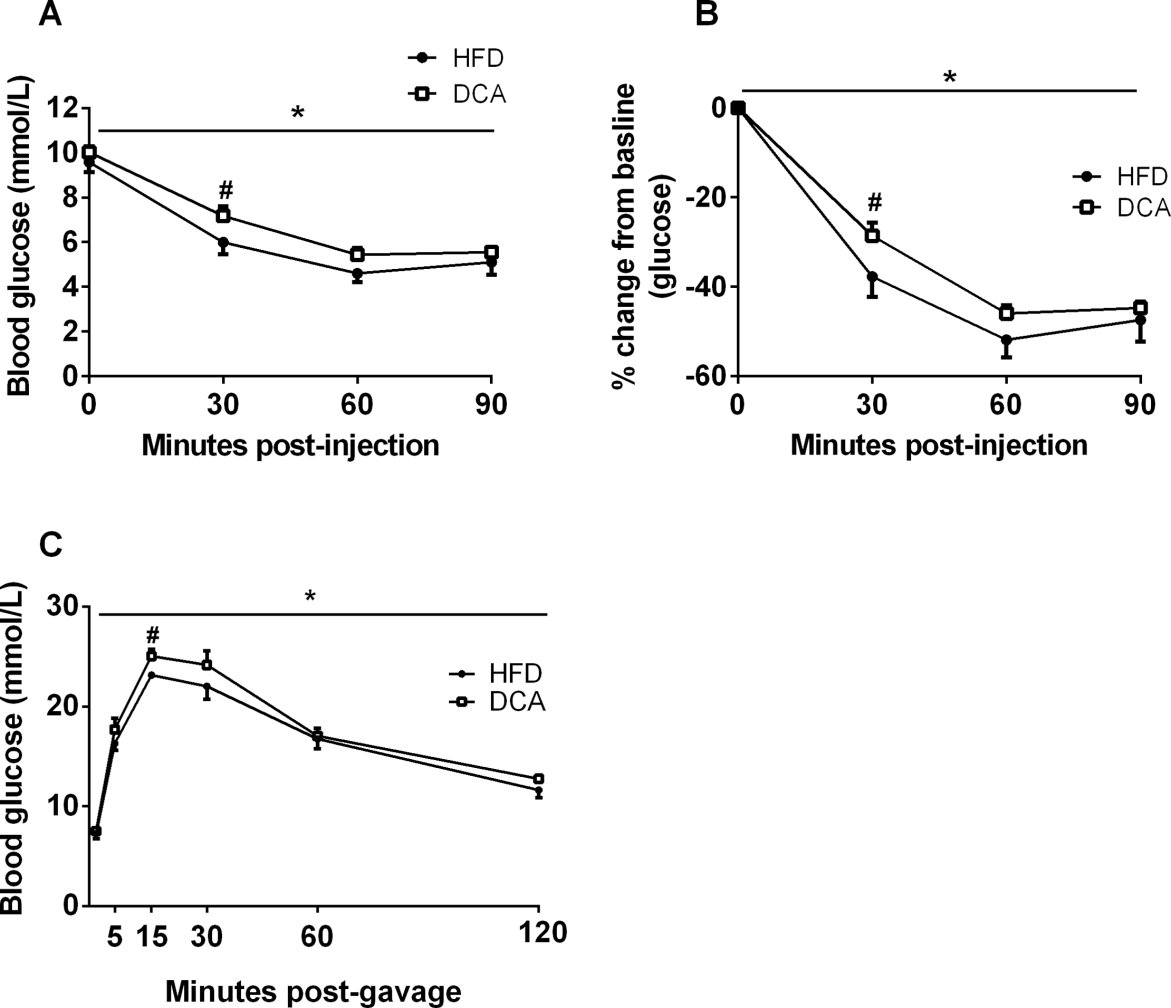
DCA supplementation impairs glucose homeostasis. Absolute blood glucose concentrations (A) and percentage change from baseline blood glucose concentrations (B) during an insulin tolerance test. (C) Blood glucose concentrations during an oral glucose tolerance test. Data are expressed as mean ± SEM, *P<0.05 by two-factor ANOVA, ^#^*P*<0.05 by Student’s t-test, *n* = 8 per group.

To determine if the effect of DCA supplementation to increase hepatic ER stress and impair hepatic insulin signaling impacts whole body glucose regulation, an ITT and an OGTT were performed in a separate cohort of mice receiving a HFD with and without DCA supplementation. Cumulative energy intake, body weight and adiposity (S2 Fig) did not differ between groups. During the ITT, absolute blood glucose concentrations were higher (Fig 4A, *P*<0.05) and the percentage change from baseline blood glucose concentrations was lower (Fig 4B, *P*<0.05) in DCA compared with HFD-fed mice. Glucose excursions were higher in DCA compared with HFD-fed mice during the OGTT (Fig 4C, *P*<0.05). Together, these data demonstrate that DCA supplementation impairs glucose homeostasis *in vivo*.

## Discussion

Herein, we provide novel data on the impact of the hydrophobic bile acid subtype, DCA, on hepatic ER homeostasis and insulin signaling. Specifically, we find that DCA increases circulating bile acid profile hydrophobicity, increases hepatic ER stress and reduces hepatic insulin signaling, independently of body weight. *In vivo* assessment of glucose and insulin tolerance demonstrated that DCA supplementation impairs glucose homeostasis, independently of body weight. Together, our data suggest that bile acid profile hydrophobicity may impact metabolic health by regulating ER homeostasis.

Bile acids have a well-defined role in lipid metabolism and an increasingly appreciated role in glucose metabolism [22, 23]. Human clinical studies point to an important role for bile acid profile in the pathophysiology of type 2 diabetes [6]; however, the mechanisms by which endogenous bile acid profile regulates glucose homeostasis remain incompletely defined. The circulating bile acid profile hydrophobicity index is elevated in patients with type 2 diabetes compared with patients with normoglycemia [6]. Furthermore, we have previously reported that a certain type of bariatric surgery, vertical sleeve gastrectomy, decreases the hydrophobicity of the circulating bile acid profile and improves glucose regulation in mice [16]. In the present study, DCA supplementation increased the hydrophobicity index of the circulating bile acid pool, which was associated with impaired hepatic ER homeostasis as well as impaired glucose regulation and hepatic insulin signaling. This is in contrast to previous work showing that treatment with the hydrophilic bile acid subtype, UDCA, decreases ER stress and improves glucose homeostasis [8]. Therefore, our data suggest that bile acid subtypes differentially regulate ER homeostasis and glucose homeostasis depending on their hydrophobicity.

The more hydrophobic a bile acid subtype is, the greater its detergent properties and ability to disrupt cell membranes [7]. In fact, hydrophobic bile acid subtypes, such as DCA, have been shown to promote inflammation and are strongly associated with the development of several types of cancer in human patients [24–28]. Furthermore, we find that vertical sleeve gastrectomy decreases the relative abundance of DCA in the circulating bile acid pool in mice which is associated with improved glucose homeostasis [16]. Hydrophilic bile acid subtypes, such as UDCA, have been described as chemical chaperones which have been suggested to improve ER homeostasis by improving intracellular osmotic balance to provide a more favorable protein folding environment [29]. Furthermore, there are several different bile acid receptors, including FXR and TGR5, which have been implicated in mediating the metabolic effects of bile acids [2]. Different bile acid subtypes exhibit different levels of affinity for these bile acid receptors [30, 31]. Therefore, it is possible that the effect of bile acids to regulate ER homeostasis may be mediated through these receptors. However, further work is needed to determine the molecular mechanisms by which bile acids regulate ER function.

Overall, this study demonstrates that DCA supplementation increases the hydrophobicity index of the circulating bile acid profile with an associated decrease in hepatic ER homeostasis and insulin signaling as well as impaired glucose homeostasis. Therefore, this study suggests that increased circulating bile acid profile hydrophobicity is associated with type 2 diabetes development by disrupting ER homeostasis. Furthermore, these results suggest that manipulating endogenous bile acid profile may be an effective target for the treatment of type 2 diabetes.

## Supporting information

S1 Table Effect of DCA supplementation on fasting serum bile acid subtype concentrations. Data are represented as mean ± SEM. *P<0.05, **P<0.01, ***P<0.001 by Student’s t-test. *n* = 6 per group. TCA, taurocholic acid; TLCA, taurolitocholic acid; HDCA, hyodeoxycholic acid; GUDCA, glycoursodeoxycholic acid; CDCA, chenodeoxycholic acid; UDCA, ursodeoxycholic acid; αω MCA, αω muricholic acid; βMCA, β-muricholic acid and Tαβ MCA, tauro-αβ muricholic acid.
(DOCX)

S2 Table Effect of DCA supplementation on fasting serum bile acid subtypes as a percentage of the total circulating bile acid pool. Data are represented as mean ± SEM. **P<0.01, ***P<0.001 by Student’s t-test. *n* = 6 per group. TCA, taurocholic acid; TLCA, taurolitocholic acid; HDCA, hyodeoxycholic acid; GUDCA, glycoursodeoxycholic acid; CDCA, chenodeoxycholic acid; UDCA, ursodeoxycholic acid; αω MCA, αω muricholic acid; βMCA, β-muricholic acid and Tαβ MCA, tauro-αβ muricholic acid.
(DOCX)

S1 Fig DCA supplementation does not impact fasting serum insulin, cholesterol or triglyceride concentrations. Fasting serum inulin (A), cholesterol (B) and triglyceride (C) concentration at baseline and three weeks after DCA supplementation. Data are expressed as mean ± SEM, *n* = 6 per group.
(DOCX)

S2 Fig DCA supplementation does not impact food intake, body weight or adiposity in mice used for assessment of in vivo glucose regulation. Energy intake (days 1–32, A), body weight (B) and adipose depot weights (subcutaneous (SQ), mesenteric (Mes), epididymal (Epi), retroperitoneal (RP) and brown adipose tissue (BAT)) (C) in mice used for ITT and OGTT tests. Data are expressed as mean ± SEM, *n* = 8 per group.
(DOCX)
