# Correction: Deoxycholic acid supplementation impairs glucose homeostasis in mice

**DOI:** 10.1371/journal.pone.0303110

**Published:** 2024-05-01

**Authors:** Karolina E. Zaborska, Seon A. Lee, Darline Garibay, Eumee Cha, Bethany P. Cummings

The authors issue this Correction to address the following errors in the original published article [1]:

- The third author’s name is misspelled. The correct spelling is: Darline Garibay.
- The Data Availability statement is incorrect. The correct statement is: The primary data underlying results in this article are available at https://dataverse.harvard.edu/dataverse/zaborskaetal.
- There is an error in the Fig 2 legend. The legend does not define all of its acronyms. Please see the complete, correct Fig 2 legend here.
- There is an error in the Fig 4 legend. The original legend specifies *n* = 8 per group, but for these experiments, the *n* value was 7–8 per group, as indicated in the underlying data at the above repository link. The correct legend is provided here.
- There is an error in S1 Table. The legend does not define all of its acronyms. Please see the complete, correct S1 Table legend here.
- There is an error in S2 Table. The fourth row was labeled “TLC”, but it reports TCDCA data. Additionally, the legend does not define all of its acronyms. Please see the complete, correct S2 Table and legend here.

**Fig 2:**
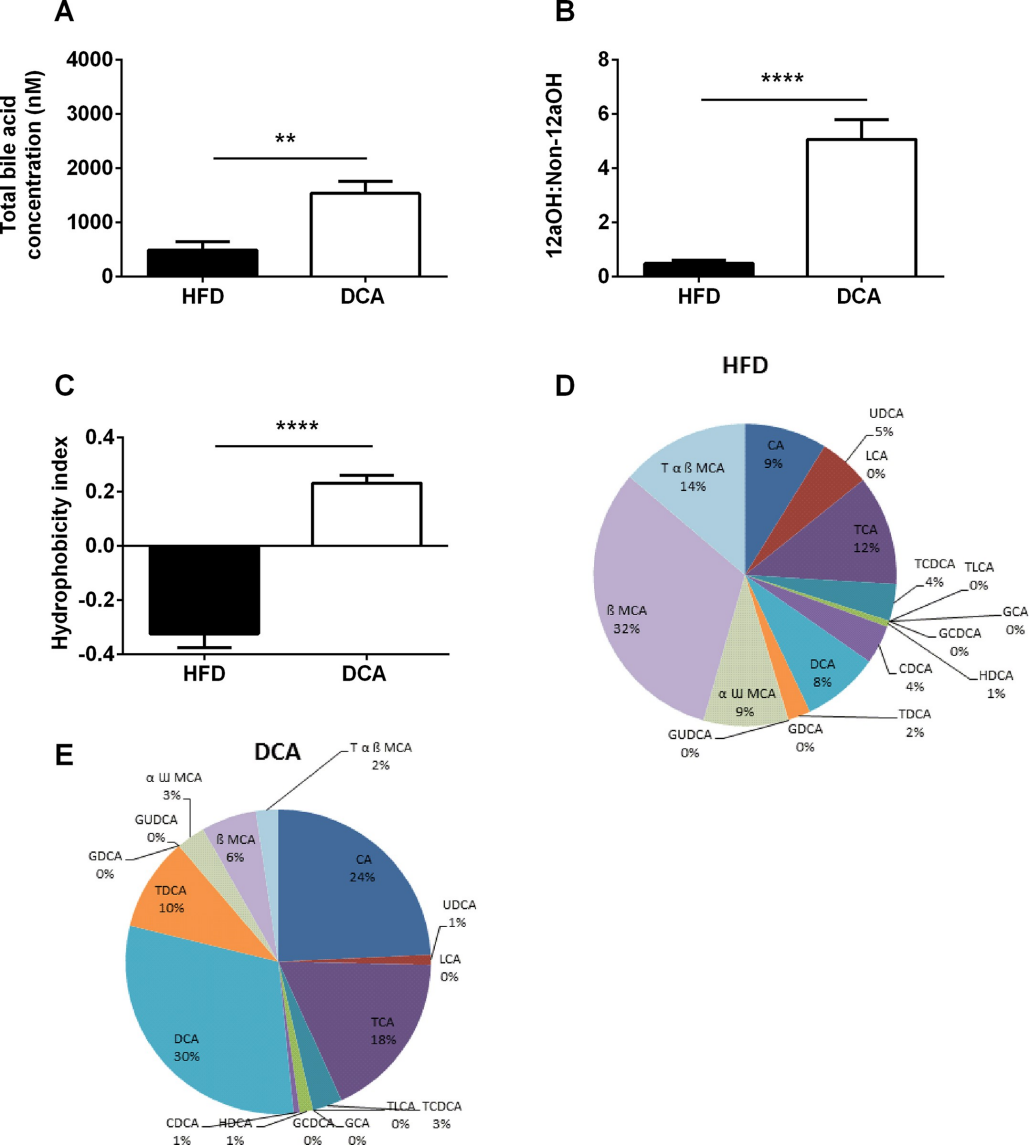
Fasting serum bile acid profile. Total bile acid concentration (A), 12αOH:non-12αOH ratio (B) and hydrophobicity index (C) in fasting serum samples. Relative proportions of bile acid subtypes in fasting serum samples from HFD (D) and DCA treated (E) mice. Data are expressed as mean ± SEM, **P<0.01, ****P<0.0001 by Student’s t-test, *n* = 6 per group. CA, cholic acid; GCA, glycocholic acid; TCA, taurocholic acid; LCA, lithocholic acid; TLCA, taurolithocholic acid; HDCA, hyodeoxycholic acid; GUDCA, glycoursodeoxycholic acid; CDCA, chenodeoxycholic acid; GCDCA, glycochenodeoxycholic acid; TCDCA, taurochenodeoxycholic acid; UDCA, ursodeoxycholic acid; DCA, deoxycholic acid; GDCA, glycodeoxycholic acid; TDCA, taurodeoxycholic acid; α ω MCA, αω muricholic acid; β MCA, β-muricholic acid and T α β MCA, tauro-αβ muricholic acid.

**Fig 4:**
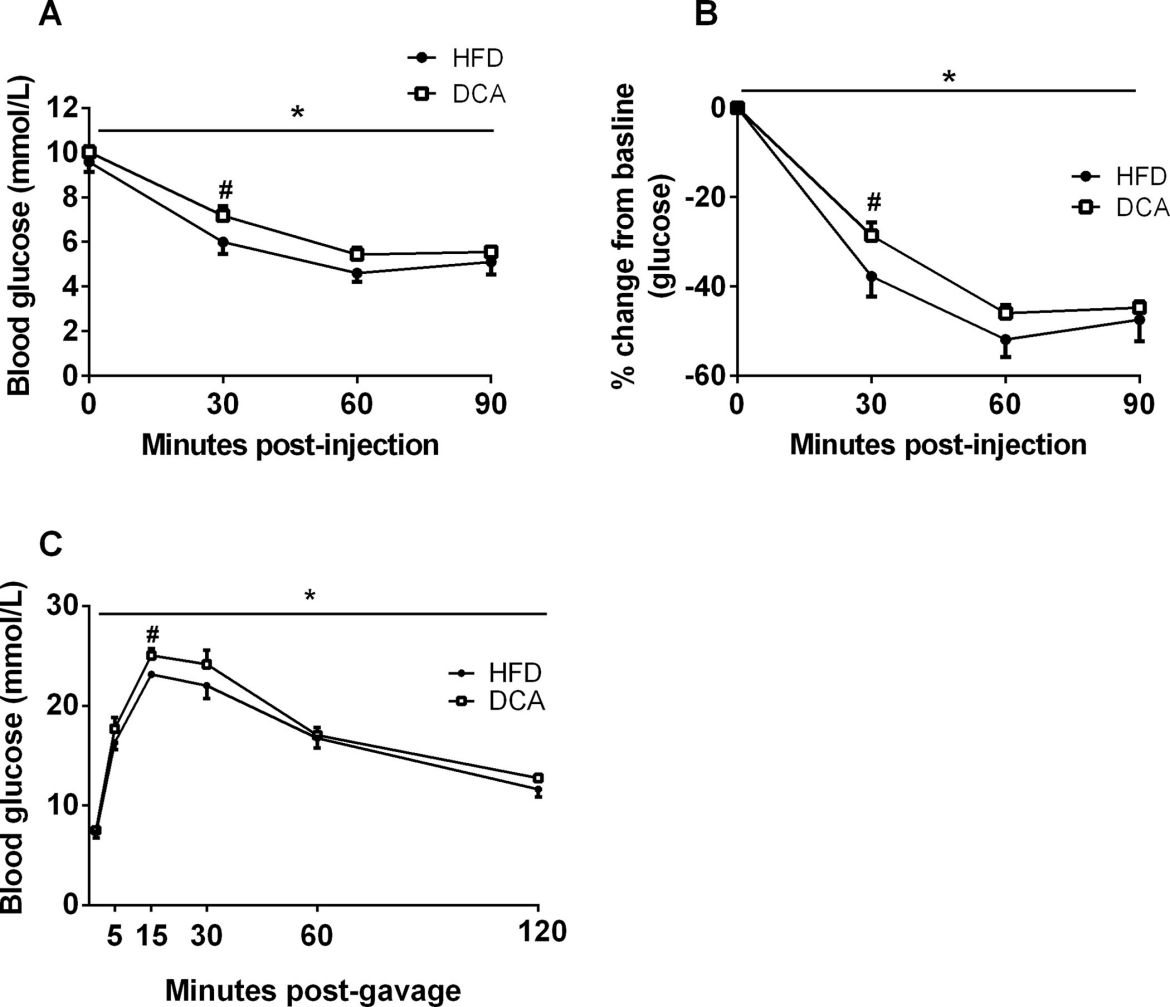
DCA supplementation impairs glucose homeostasis. Absolute blood glucose concentrations (A) and percentage change from baseline blood glucose concentrations (B) during an insulin tolerance test. (C) Blood glucose concentrations during an oral glucose tolerance test. Data are expressed as mean ± SEM, *P<0.05 by two-factor ANOVA, #*P*<0.05 by Student’s t-test, *n* = 7–8 per group.

## Supporting information

S1 Table Effect of DCA supplementation on fasting serum bile acid subtype concentrations. Data are represented as mean ± SEM. *P<0.05, **P<0.01, ***P<0.001 by Student’s t-test. *n* = 6 per group. CA, cholic acid; TCA, taurocholic acid; TCDCA, taurochenodeoxycholic acid; DCA, deoxycholic acid; TDCA taurodeoxycholic acid; HDCA, hyodeoxycholic acid; CDCA, chenodeoxycholic acid; UDCA, ursodeoxycholic acid; αω MCA, αω muricholic acid; β MCA, β-muricholic acid and T αβ MCA, tauro-αβ muricholic acid.
(DOCX)

S2 Table Effect of DCA supplementation on fasting serum bile acid subtypes as a percentage of the total circulating bile acid pool. Data are represented as mean ± SEM. **P<0.01, ***P<0.001 by Student’s t-test. *n* = 6 per group. CA, cholic acid; TCA, taurocholic acid; TCDCA, taurochenodeoxycholic acid; DCA, deoxycholic acid; TDCA taurodeoxycholic acid; HDCA, hyodeoxycholic acid; CDCA, chenodeoxycholic acid; UDCA, ursodeoxycholic acid; αω MCA, αω muricholic acid; β MCA, β-muricholic acid and T αβ MCA, tauro-αβ muricholic acid.
(DOCX)
