# Supplementary material for: Deoxycholic acid supplementation impairs glucose homeostasis in mice
Source: PLoS One. 2018 Jul 30;13(7):e0200908. doi: 10.1371/journal.pone.0200908 (PMC6066200; doi:10.1371/journal.pone.0200908)
Supplement: S1 Table — Data are represented as mean ± SEM. *P<0.05, **P<0.01, ***P<0.001 by Student’s t-test. n = 6 per group. TCA, taurocholic acid; TLCA, taurolitocholic acid; HDCA, hyodeoxycholic acid; GUDCA, glycoursodeoxycholic acid; CDCA, chenodeoxycholic acid; UDCA, ursodeoxycholic acid; αω MCA, αω muricholic acid; βMCA, β-muricholic acid and Tαβ MCA, tauro-αβ muricholic acid. (DOCX) [file pone.0200908.s001.docx]

|  | **HFD (nM)** | **DCA (nM)** |
| --- | --- | --- |
| CA | 42.9 ± 13.6 | 361.9 ± 81.5^**^ |
| UDCA | 20.7 ± 4.6 | 14.7 ± 4.4 |
| TCA | 73.0 ± 45.6 | 305.7 ± 138.1 |
| TCDCA | 16.6 ± 6.6 | 50.9 ± 16.0 |
| HDCA | 6.3 ± 4.0 | 20.3 ± 3.3^*^ |
| CDCA | 14.9 ± 2.4 | 8.9 ± 2.6 |
| DCA | 32.9 ± 7.9 | 446.2 ± 85.1^***^ |
| TDCA | 14.1 ± 9.4 | 159.3 ± 41.1^**^ |
| αω MCA | 39.6 ± 11.9 | 42.8 ± 10.5 |
| β MCA | 155.7 ± 58.9 | 83.6 ± 19.7 |
| T αβ MCA | 70.1 ± 24.8 | 39.6 ± 14.7 |

**Supplementary table 1.** **Effect of DCA supplementation on fasting serum bile acid subtype concentrations**. Data are represented as mean ± SEM. **P*<0.05, ***P*<0.01, ****P*<0.001 by Student’s t-test. *n* = 6 per group. TCA, taurocholic acid; TLCA, taurolitocholic acid; HDCA, hyodeoxycholic acid; GUDCA, glycoursodeoxycholic acid; CDCA, chenodeoxycholic acid; UDCA, ursodeoxycholic acid; αω MCA, αω muricholic acid; βMCA, β-muricholic acid and Tαβ MCA, tauro-αβ muricholic acid.
