# Supplementary material for: Deoxycholic acid supplementation impairs glucose homeostasis in mice
Source: PLoS One. 2018 Jul 30;13(7):e0200908. doi: 10.1371/journal.pone.0200908 (PMC6066200; doi:10.1371/journal.pone.0200908)
Supplement: S2 Table — Data are represented as mean ± SEM. **P<0.01, ***P<0.001 by Student’s t-test. n = 6 per group. TCA, taurocholic acid; TLCA, taurolitocholic acid; HDCA, hyodeoxycholic acid; GUDCA, glycoursodeoxycholic acid; CDCA, chenodeoxycholic acid; UDCA, ursodeoxycholic acid; αω MCA, αω muricholic acid; βMCA, β-muricholic acid and Tαβ MCA, tauro-αβ muricholic acid. (DOCX) [file pone.0200908.s002.docx]

|  | **HFD (%)** | **DCA (%)** |
| --- | --- | --- |
| CA | 8.81 ± 0.52 | 24.28 ± 4.78^**^ |
| UDCA | 5.40 ± 0.89 | 1.03 ± 0.28^***^ |
| TCA | 11.75 ± 3.78 | 17.88 ± 6.88 |
| TLC | 3.94 ± 0.82 | 3.16 ± 0.68 |
| HDCA | 0.67 ± 0.43 | 1.40 ± 0.19 |
| CDCA | 4.13 ± 0.78 | 0.64 ± 0.19^**^ |
| DCA | 8.25 ± 1.26 | 30.43 ± 4.46^***^ |
| TDCA | 2.41 ± 0.81 | 9.92 ± 2.04^**^ |
| αω MCA | 9.02 ± 1.64 | 3.06 ± 0.83^**^ |
| β MCA | 31.83 ± 4.82 | 5.85 ± 1.36^***^ |
| T αβ MCA | 13.75 ± 1.34 | 2.35 ± 0.52^***^ |

**Supplementary table 2.** **Effect of DCA supplementation on fasting serum bile acid subtypes as a percentage of the total circulating bile acid pool**. Data are represented as mean ± SEM. ***P*<0.01, ****P*<0.001 by Student’s t-test. *n* = 6 per group. TCA, taurocholic acid; TLCA, taurolitocholic acid; HDCA, hyodeoxycholic acid; GUDCA, glycoursodeoxycholic acid; CDCA, chenodeoxycholic acid; UDCA, ursodeoxycholic acid; αω MCA, αω muricholic acid; βMCA, β-muricholic acid and Tαβ MCA, tauro-αβ muricholic acid.
