# Supplementary material for: Deoxycholic acid supplementation impairs glucose homeostasis in mice
Source: PLoS One. 2018 Jul 30;13(7):e0200908. doi: 10.1371/journal.pone.0200908 (PMC6066200; doi:10.1371/journal.pone.0200908)
Supplement: S1 Fig — Fasting serum inulin (A), cholesterol (B) and triglyceride (C) concentration at baseline and three weeks after DCA supplementation. Data are expressed as mean ± SEM, n = 6 per group. (DOCX) [file pone.0200908.s003.docx]

**Supplementary Figure 1. DCA supplementation does not impact fasting serum insulin, cholesterol or triglyceride concentrations.** Fasting serum inulin (A), cholesterol (B) and triglyceride (C) concentration at baseline and three weeks after DCA supplementation. Data are expressed as mean ± SEM, *n*=6 per group.
