# Supplementary material for: Deoxycholic acid supplementation impairs glucose homeostasis in mice
Source: PLoS One. 2018 Jul 30;13(7):e0200908. doi: 10.1371/journal.pone.0200908 (PMC6066200; doi:10.1371/journal.pone.0200908)
Supplement: S2 Fig — Energy intake (days 1–32, A), body weight (B) and adipose depot weights (subcutaneous (SQ), mesenteric (Mes), epididymal (Epi), retroperitoneal (RP) and brown adipose tissue (BAT)) (C) in mice used for ITT and OGTT tests. Data are expressed as mean ± SEM, n = 8 per group. (DOCX) [file pone.0200908.s004.docx]

**Supplementary Figure 2. DCA supplementation does not impact food intake, body weight or adiposity in mice used for assessment of *in vivo* glucose regulation.** Energy intake (days 1-32, A), body weight (B) and adipose depot weights (subcutaneous (SQ), mesenteric (Mes), epididymal (Epi), retroperitoneal (RP) and brown adipose tissue (BAT)) (C) in mice used for ITT and OGTT tests. Data are expressed as mean ± SEM, *n*=8 per group.
